# Nondisclosure of queer identities is associated with reduced scholarly publication rates

**DOI:** 10.1371/journal.pone.0263728

**Published:** 2022-03-02

**Authors:** Joey Nelson, Allison Mattheis, Jeremy B. Yoder

**Affiliations:** 1 Office of the Vice Provost for Undergraduate Education, Stanford University, Stanford, California, United States of America; 2 Division of Applied and Advanced Studies in Education, California State University, Los Angeles, Los Angeles, California, United States of America; 3 Department of Biology, California State University Northridge, Northridge, California, United States of America; Universita degli Studi di Napoli Federico II, ITALY

## Abstract

Nondisclosure of lesbian, gay, bisexual, transgender, asexual, or otherwise queer (LGBTQA) identities in the workplace is both common and stressful to those who do not disclose. However, we lack direct evidence that nondisclosure of LGBTQA identity affects worker productivity. In two surveys of LGBTQA-identified scientists, we found that those who did not disclose LGBTQA identities in professional settings authored fewer peer-reviewed publications—a concrete productivity cost. In the second survey, which included straight and cisgender participants as a comparison group, we found that LGBTQA participants who disclosed their sexual orientation had publication counts more like non-LGBTQA participants than those who did not disclose, and that all three groups had similar time since first publication given their academic career stage. These results are most consistent with a productivity cost to nondisclosure of LGBTQA identity in professional settings, and suggest a concrete need to improve scientific workplace climates for sexual and gender minorities.

## Introduction

Lesbian, gay, bisexual, transgender, asexual, or otherwise queer (LGBTQA) identities are not necessarily evident without deliberate disclosure. This disclosure is popularly represented as a discrete “coming out,” though revelation and expression of queer identity is more often a continuous process [1–4]. Free expression of queer identities is generally understood to be affirming and liberating, but choosing to come out also remains a decision with potentially grave consequences, particularly in professional settings [5–9]. In the United States, Federal law has only recently been interpreted to prohibit employment discrimination based on sexual orientation, gender identity, and transgender status [10, 11], and this progress remains fragile and incomplete [12, 13]. LGBTQ-identified individuals continue to face barriers across society, including in healthcare [14, 15], housing [16], and economic and social services [17–19]. Even when their identities are legally protected, LGBTQA-identifying individuals may fear that coming out will have negative consequences for their relationships with colleagues [20–22]. Disclosure of queer identities in professional settings thus risks career disadvantages, discriminatory treatment, or worse [6, 9, 23, 24].

At the same time, nondisclosure of queer identities in any context has been shown to be a source of stress and distraction for nondisclosing individuals [4, 20, 21, 25, 26], and nondisclosure in the workplace reduces job satisfaction, social integration with colleagues, and identification with employers [8, 23, 27–29]. Proponents of anti-discrimination protections for minority sexual orientation and gender identities have therefore argued that ensuring the freedom to express queer identities openly can promote employee productivity [28, 30]. Studies using surveys or interviews have differed over whether disclosure of queer identities leads to greater comfort in professional settings [1, 8, 22, 27, 28], however, and no study to date has directly tested whether nondisclosure is associated with reduction in any concrete measure of productivity.

Scientific research careers offer a unique opportunity to examine this relationship. Particularly in academic science and technical fields, research productivity is widely judged by a fairly straightforward metric: the number of peer-reviewed articles a researcher publishes. Peer-reviewed publication counts are at best a crude metric of impactful or meaningful scholarly work, as scholars in most disciplines and workplace contexts also contribute in the form of teaching, mentoring, outreach, administrative service, and even activism—to name just a few possibilities. Nevertheless, successful completion of publishable research papers is prioritized in graduate training for most scientific fields, and publication activity informs hiring, promotion, and tenure decisions for academic research faculty [31–33]. Publication counts have been used in prior studies to directly quantify systematic disadvantages to women [31, 34, 35] and people of color [36] in academic careers. These previous studies suggest that academic publishing rates reflect broader issues regarding the impact of systematic racism and sexism on productivity, career advancement, and longevity in the field. Gender discrimination has close links to discrimination against queer identities: greater representation of women among workers in STEM fields is positively associated with LGBTQA-identifying workers’ disclosure of their identities in professional settings [1], and with greater representation of men in same-sex relationships [37].

Gender discrimination, including bias in peer review, can result in higher publication rates for men relative to women and create pressure for women to publish more than men to achieve similar status [38]. Some studies have found that although publication rates are more similar at early career stages, women tend to have shorter careers in science than men and higher rates of dropout or career transition, resulting in lower overall productivity [35, 39, 40]. Other research has found that scholars from minoritized groups have fewer opportunities to publish at the Ph.D. and postdoctoral levels [41], and that women achieve tenure at lower rates than men even with similar publication records [42]. Moreover, gender gaps persist in authorship of publications, especially in prestigious journals, across science, technology, engineering, mathematics, and medicine [43], and sociocultural expectations of gender roles impact scientific publishing rates and career advancement [44, 45]. Although important conclusions have been drawn from the aggregate of work examining gender gaps and discrimination in academic publishing, many of these studies have relied on author name as a proxy for gender, and assume a man/woman gender binary. The literature on gender gaps in scholarly publication has not, to our knowledge, explicitly examined gender identities beyond the man/woman binary, and has not accounted for transgender or questioning individuals. To avoid the limitations of mapping names onto a gender binary, we argue for an understanding of gender as more expansive than a binary, and for methodological approaches in which study participants self-identify aspects of their identity, including gender, whenever possible.

The possibility that disclosure or nondisclosure of queer identities might have a detectable impact on scholarly publication rates is a logical extension of the broader research linking publication rates to the experiences of women and other minoritized identities in STEM careers. We hypothesize that the increased stress and decreased sense of workplace belonging associated with nondisclosure of queer identities creates its own kind of systematic disadvantage that should manifest in academic publication rates, and that disclosure of queer identities in professional settings may offset or eliminate this disadvantage. It is possible that differences in publication rates could be associated with disclosure of LGBTQA identities if individuals who publish more frequently feel more secure in their jobs, and thereby better able to disclose minoritized identities. However, if this is the case, we would expect that disclosing LGBTQA individuals would publish at rates higher than their straight cisgender colleagues, and achieve key promotions (completion of a PhD, hiring as a postdoc, hiring as faculty, and advancement to tenure) earlier. In the broader context of institutional sexism in academia, we further expect that the effect of disclosure may interact with gender identity, gender expression, and cisgender or transgender status.

Specifically, we hypothesize that

1. LGBTQA-identified scientists will have reduced publication rates relative to straight-identified, cisgender scientists; but disclosing LGBTQA-identified scientists will have publication rates more like those of their straight cisgender peers.
2. Disclosing LGBTQA-identified scientists will not have achieved key career stages earlier than nondisclosing LGBTQA-identified scientists, or than straight cisgender scientists.
3. Disclosure or nondisclosure of gender identity and/or transgender status, which directly confronts an individual’s status with respect to institutional sexism, will not have the same effect on productivity as disclosure of sexual orientation. Rather, scientists who identify as women, nonbinary, agender, or otherwise non-male will show reduced publication rates relative to men regardless of disclosure status; and individuals whose gender expression is other than masculine will show reduced publication rates relative to those with masculine gender expression.

Here, we test these hypotheses with responses to two surveys of queer academics in science, technology, engineering, and mathematics (STEM), which relate disclosure of queer identities in professional settings to publication counts. In both surveys, we find that nondisclosure is associated with reduced publication counts. In the second survey, which included both LGBTQA-identified participants and straight cisgender participants, we find that LGBTQA-identified participants who disclosed their identities in professional settings had publication counts more like those of straight cisgender participants. We further find that disclosing LGBTQA, nondisclosing LGBTQA, and straight cisgender participants at do not differ in their time since first publication within discrete career stages, consistent with differences in publication rate arising from a cost of nondisclosure, rather than security afforded by productivity. Finally, we compare the effect of nondisclosure to the effects of LGBTQA identity apart from disclosure, participants’ self-described gender expression, and participants’ ratings of the “climate” for LGBTQA-identified individuals in their workplaces, as well as to the possible confounding effects of increasing publication count with career progress. We find that a negative effect of nondisclosure is robust to these other explanatory factors.

## Results

Language to describe sexual orientation, gender identities, and transgender experiences is complex and changeable in usage across communities, among individuals, and over time. Here, we use “queer” to refer inclusively to individuals identifying as other than exclusively cisgender and straight, and variants of the initialism “LGBTQA” to refer to sets of specific identities (e.g., LBQ for lesbian, bisexual, and otherwise queer) when applicable, particularly in discussing prior works focused on specific queer identities. We use the word “straight” as opposed to “heterosexual” largely to parallel our inclusive and colloquial usage of “queer”. We further follow a recent proposal to clearly differentiate gender identity (whether an individual is a man, a woman, nonbinary, agender, or other gender) from transgender or cisgender status, or “gender modality” (whether an individual’s gender identity aligns with the gender they were assigned at birth; [46]), though this differentiation was not formally proposed or commonly used at the time of our survey design and data collection. We use “nonbinary” inclusively of gender identities beyond the binary of men/women, while acknowledging that this simplifies gender diversity, including agender and genderqueer identities. Finally, we describe expression of queer identities in terms of “disclosure” or “nondisclosure” to emphasize that revealing a queer identity is often an intentional act, and that a person’s choice not to disclose their queer identity in a particular context does not mean they are deceiving anyone. LGBTQA-identified individuals who do not disclose queer identity may be assumed to be straight or cisgender or to have binary gender identity as a majoritarian default, as has been reported in STEM workplaces [47]. At all points we do our best to follow the self-descriptions of participants in our surveys. We discuss our approach to translating survey participants’ open-ended descriptions of their identities into simplified categories for analysis in more depth in Methods, below.

### The 2013 queer in STEM survey

In 2013 we recruited LGBTQA-identified professionals in STEM careers to complete an online survey about their career progress and workplace experiences (ref [1]; survey design and participant recruitment briefly described in the Methods, specific survey items in S1 File). We asked participants to report the number of peer-reviewed papers they had published, to describe their gender identity and cisgender or transgender status, and to describe their current job or career position. We also asked participants to rate their disclosure of their queer identities in professional contexts using a scale from 0 (“I am not out to anyone in this group”) to 5 (“As far as I’m aware, everyone in this group could know”). We classified participants reporting disclosure scores below the midpoint (“Less than half of the people in this group know”) as “nondisclosing” in professional settings, and those reporting greater degrees of openness as “disclosing”.

**Fig 1:**
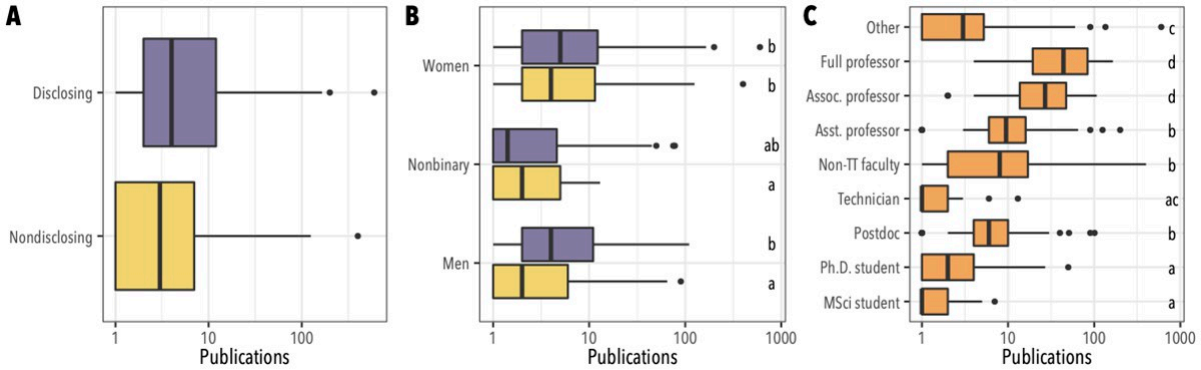
Factors associated with differences in publication counts reported in the 2013 survey. (**A**) Publication count differs significantly by whether or not participants disclosed their LGBTQA identities in professional settings (two-sided t-test, p < 0.001). (**B**) Publication count by gender identity (women; nonbinary, agender, or otherwise beyond the binary; men) and disclosure of LGBTQA identities, colored as in (A). (**C**) Publication count by academic position. In all panels, boxes give 25^th^, 50^th^, and 75^th^ quantiles; whiskers reach to 1.5x interquartile range; in (B) and (C) letters indicate groups that differ significantly with p < 0.05 in a Tukey HSD test.

Among 633 participants who were working at any stage of an academic career and who had authored at least one peer-reviewed publication, those who did not disclose their queer identities in professional settings also reported having authored significantly fewer peer-reviewed publications (Fig 1A; mean ± SE publications for nondisclosing participants = 8.5 ± 1.7, for disclosing = 15.1 ± 2.1; p < 0.001, two-tailed t-test on log-transformed data). Publication counts also differed by gender identities—whether participants identified as men, women, or nonbinary or agender. This interacted with disclosure such that the difference in publication counts between disclosing and nondisclosing participants was significant for queer men, but not for queer women or nonbinary participants (Fig 1B; mean ± SE publications for nondisclosing men = 4.9 ± 0.8, for disclosing men = 12.2 ± 1.8, for nondisclosing nonbinary participants = 3.4 ± 0.6, for disclosing nonbinary participants = 10 ± 3.7, for nondisclosing women = 15.8 ± 4.6, for disclosing women = 17.9 ± 3.7; two-way ANOVA p < 10^−4^ for identity, p < 0.01 for disclosure, p = 0.02 for the interaction). Publication counts were not significantly explained by participants’ ratings of their current workplaces as welcoming or unwelcoming to queer individuals (S1 Fig; one-way ANOVA p = 0.11); or by their STEM fields (S2 Fig; one-way ANOVA p = 0.13). However, there was also an expected, and strong, effect of participants’ job positions, with those in more senior positions reporting more publications (Fig 1C; one-way ANOVA p < 10^−6^). Disclosure differed among positions (Χ^2^_df=8_ test, p < 10^−5^) such that participants in more senior positions were also more likely to disclose queer identities (S3 Fig; roughly 51% of Ph.D. students nondisclosing, versus 12% of full professors); and a two-way ANOVA with position and openness found a significant effect for position (p < 10^−6^) but not disclosure (p = 0.87). Thus, in the 2013 survey data the effect of nondisclosure is fully confounded with seniority—they are both consistent with the possibility that nondisclosure has a negative effect on productivity, and the possibility that more senior scientists, who have published more, are more likely to disclose LGBTQA identities.

### The 2016 queer in STEM survey

To better address the question of how nondisclosure of queer identity may impact productivity, we developed and conducted a second survey in 2016. In the 2016 survey we again asked participants to describe the climates of their workplaces, and to report their STEM fields, job titles, and number of peer-reviewed publications authored (item text in S1 File). Rather than ask about disclosure of queer identities as a whole, we asked participants to separately rate their openness in professional settings in relation to their sexual orientation and in relation to their “gender identity”—following terminology in use at the time and familiar to expected survey participants, we defined this to include both gender identity and gender modality (see item text, S1 File). In addition, to better control for the confounding effect of seniority, we asked participants how many years had elapsed since the publication of their first peer-reviewed paper. Finally, to provide an additional control for the effect of nondisclosure of queer identities, we recruited cisgender straight scientists as well as queer-identified scientists.

**Fig 2:**
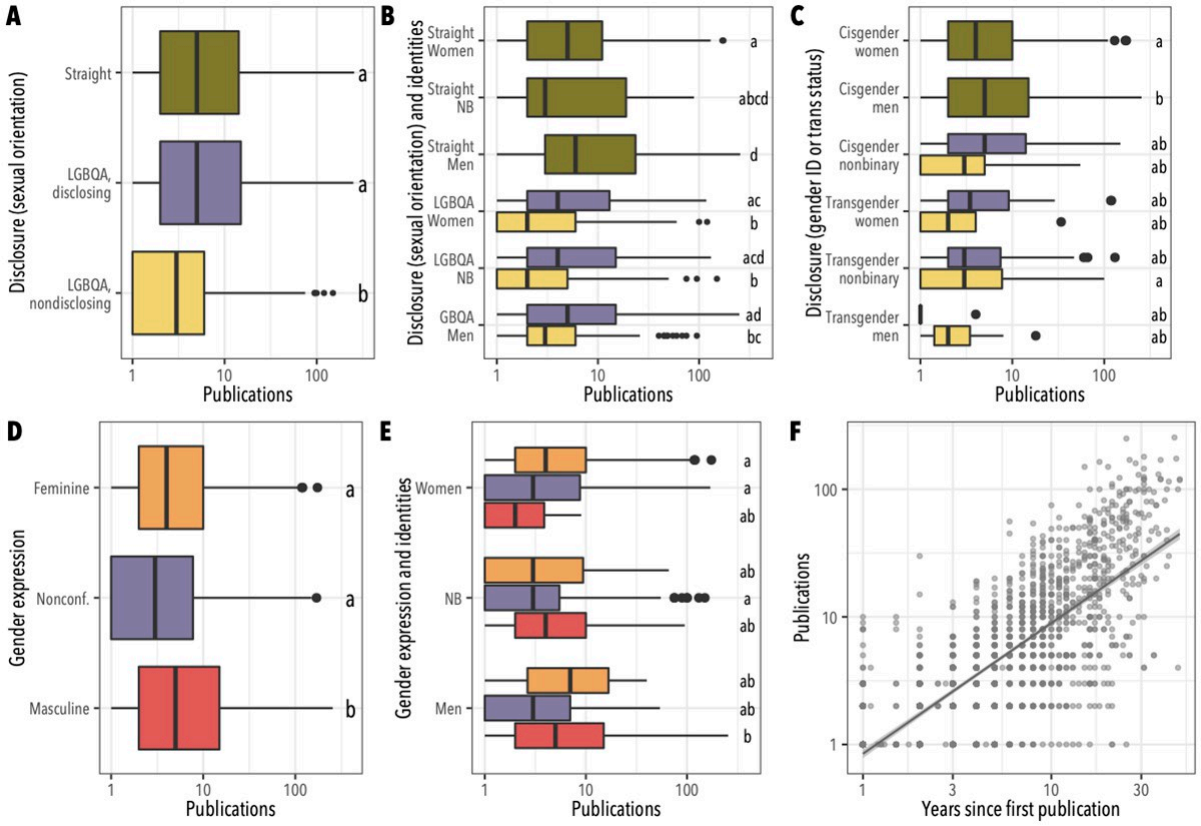
Factors associated with differences in publication counts reported in the 2016 survey. (**A**) Publication counts by whether or not participants disclosed their sexual orientation in professional settings. (**B**) Publication counts by gender identity (as in Fig 1B) as well as openness about sexual orientation, with color-coding as in (A). (**C**) Publication counts by participants’ gender identity and cisgender or transgender status, and disclosure of gender identity or trans status. (**D**) Publication counts by participants’ gender expression. (**E**) Publication counts by gender identity and gender expression, with color-coding as in (D). Boxplot interpretation as in Fig 1. (**F**) Scatterplot of publication count versus time since first publication; line and shaded band indicate linear regression ± standard error.

A total of 1,116 LGBTQA-identified and 629 cisgender straight survey participants worked in academic settings and had authored at least one peer-reviewed paper. Across all LGBQA participants, those who said they did not disclose their sexual orientation in professional settings reported fewer peer-reviewed papers compared to those who disclosed, or to straight participants (Fig 2A; mean ± SE publications for nondisclosing LGBQA participants = 7.1 ± 0.5, for disclosing LGBQA participants = 13.9 ± 1.2, and for straight participants = 15.6 ± 1.1; Tukey HSD test p < 10^−6^ for nondisclosing LGBQA versus disclosing LGBQA and for nondisclosing LGBQA versus straight; p = 0.33 for disclosing queer versus straight). We found broadly similar patterns when considering disclosure of sexual orientation based on gender identities separately: among GBQA men, LGBQA nonbinary participants, and LGBQA women, those who did not disclose their sexual orientation had significantly fewer publications (Fig 2B; mean ± SE for disclosing GBQA men = 14.7 ± 1.8, for nondisclosing GBQA men = 7.7 ± 0.9, for disclosing LGBQA nonbinary participants = 15.2 ± 3.0, for nondisclosing LGBQA nonbinary participants = 7.3 ± 1.5, for disclosing LGBQA women = 11.7 ± 1.8, for nondisclosing LGBQA women = 6.6 ± 0.7; Tukey HSD p < 0.05 in all cases). We also found that straight women, disclosing and nondisclosing queer women, nondisclosing men, and nondisclosing nonbinary participants all reported fewer publications than straight men (Fig 2B; mean ± SE publications for straight women = 12.1 ± 1.1, for straight men = 21.0 ± 2.3; Tukey HSD p < 0.05 in all cases); while straight women reported publication counts not significantly different from straight nonbinary participants or any queer participants who disclosed their sexual orientation (Fig 2B; Tukey HSD p > 0.99 in all cases).

In addition to factors paralleling those addressed in the 2013 survey, we asked participants in the 2016 survey to rate their openness about their gender identity in professional settings (following colloquial terminology, the item text defined “gender identity” to encompass both gender identity as men, women, nonbinary, agender, or genderqueer; and gender modality, or cisgender or transgender status [46]; see S1 File). We also asked participants to describe their gender expression as masculine, feminine, androgynous, or terms of their choosing. This allowed us to address whether gender identity and expression mediated effects associated with queer identity and disclosure. Disclosure of gender identity and cisgender or transgender status did have a significant effect on publication count (one-way ANOVA, p = 0.002); but the difference between disclosing and nondisclosing individuals, considered within either nonbinary cisgender participants, transgender men, transgender women, or transgender nonbinary participants, was not greater than expected by chance (Fig 2C; mean ± SE for disclosing cis nonbinary = 15.1 ± 5.2, for nondisclosing cis nonbinary = 5.6 ± 1.8, for disclosing trans men = 1.5 ± 0.5, for nondisclosing trans men = 4.1 ± 1.5, for disclosing trans women = 22.1 ± 11.1, for nondisclosing trans women = 8.4 ± 6.4, for disclosing trans nonbinary = 11.1 ± 3.2, for nondisclosing trans nonbinary = 10.4 ± 1.8, for cis men = 15.2 ± 1.1, for cis women = 9.7 ± 0.6; Tukey HSD p < 0.01 for cis men versus cis women and for cis men versus trans nonbinary nondisclosing, and p > 0.05 for all other comparisons). Gender expression did have a significant effect: participants whose self-described gender expression was feminine or nonconforming to the gender binary reported significantly fewer publications than participants whose gender expression was masculine (Fig 2D; mean ± SE publications for masculine participants = 15.2 ± 1.1, for nonconforming participants = 10.3 ± 1.1, for feminine participants = 9.7 ± 0.7; Tukey HSD test p < 0.0001 for masculine versus each other group). These differences associated with gender expression did not translate to significant differences within gender identities, however (Fig 2E; two-way ANOVA p < 10^−5^ for gender expression, p = 0.38 for gender identity, p = 0.34 for the interaction; Tukey HSD test p > 0.05 in all cases). As in the 2013 survey, we found variation in publication count was not significantly associated with participants’ ratings of their workplace climates (S4 Fig; one-way ANOVA p = 0.8) or by STEM field (S5 Fig; one-way ANOVA p = 0.21), but there was a strong, positive association between time since the publication of participants’ first peer-reviewed paper and the total number of papers they had published (Fig 2F; product-moment correlation on log-transformed data = 0.76, p < 10^−6^).

An association between publication count and disclosure could arise because disclosure facilitates productivity—if disclosure reduces job-related stress and increases workplace satisfaction. The same association could also arise because productivity facilitates disclosure—if more productive individuals feel more secure in taking the risks associated with disclosure. In the latter case, we might expect disclosing queer participants to have *higher* publication rates than straight participants, rather than matching them (Fig 2A and 2B). Nevertheless, to more fully understand the causal direction of the association between publication count and disclosure, we also examined time since first publication for different identity groupings within academic career stages (i.e., job position within the academic career progression from graduate student to full professor). If queer participants are more likely to disclose their queer identity when they feel secure as a result of career accomplishment, we might expect them to show evidence of faster academic career advancement. That is, within a given academic career stage, the time elapsed since first publication by queer disclosing participants would be shorter than for both cisgender-straight and queer nondisclosing participants. However, we found no significant difference in time since first publication among identity groups (straight, LGBQA disclosing sexual orientation, and LGBQA nondisclosing) within academic ranks (S6 Fig; Tukey HSD p > 0.05 for all comparisons within career stage). This lack of differences in the timing of career advancement suggests that the underlying cause of the association between disclosure and publication rates is more likely the cost of nondisclosure to LGBTQA-identified individuals, as discussed above, rather than disclosure becoming available to more productive LGBTQA-identified individuals.

Sexual orientation, gender identity, gender modality, and gender expression are distinct components of identity, and they interact in complex ways (see Methods discussion of our classifications based on participants’ self-descriptions). To ascertain which of these factors showing significant association with variation in publication count in the 2016 survey contributed most strongly, we therefore used a model comparison approach. We fitted linear models using additive and interacting combinations of time since first publication (log-transformed), disclosure of sexual orientation, disclosure of gender identity or transgender status, gender expression, sexual orientation, gender identity, and gender modality to log-transformed publication counts, and compared model fit in terms of corrected Akaike Information Criterion (AICc; [48]) scores (Table 1). The best-fit model explained variation in publication count with additive effects of disclosure of sexual orientation and time since first publication, and an interaction between the two (adjusted R^2^ = 0.59, AICc = 1379.9); a model predicting publication count with the interacting effects of disclosure of sexual orientation and time had ΔAICc = 3.9, indicating worse fit; all other models had ΔAICc ≥ 6.8, indicating substantially worse fit (Table 1).

**Table 1:** Variation explained and model fit for linear models predicting log-transformed publication counts with elements of queer identity and disclosure.

Model^a^	df	Adj. R^2^	AICc	ΔAICc
D: time	7	0.58	1383.8	3.9
D + time	5	0.58	1394.4	14.4
**DOri: time**	**19**	**0.59**	**1379.9**	**0.0**
DOri + time	11	0.58	1386.7	6.8
G: time	7	0.57	1424.5	44.6
G + time	5	0.57	1420.7	40.8
DGen: time	21	0.58	1417.6	37.7
DGen + time	12	0.58	1411.4	31.4
GE: time	7	0.58	1402.2	22.3
GE + time	5	0.58	1405.5	25.6
GIO: time	13	0.58	1394.8	14.9
GIO + time	8	0.58	1394.1	14.2
GIM: time	13	0.58	1409.0	29.1
GIM + time	8	0.58	1406.1	26.1
D	10	0.05	2799.2	1419.3
DOri	4	0.06	2807.6	1427.7
G	11	0.06	2871.6	1491.7
DGen	4	0.02	2889.1	1509.2
GE	4	0.04	2871.2	1491.3
GIO	7	0.02	2833.0	1453.1
GIM	7	0.02	2869.2	1489.3
time	3	0.57	1420.0	40.1

Predictor variables are: disclosure of sexual orientation (D, grouping as in Fig 2A), queer identity and disclosure of sexual orientation (DOri, grouping as in Fig 2B), disclosure of gender identity (G), gender identity and cisgender or transgender status and disclosure of gender identity or cisgender or transgender status (DGen, groupings as in Fig 2C), gender expression (GE, groupings as in Fig 2D), gender identity and orientation (GIO), gender identity and gender modality (GIM), and time since first publication (time).

**Fig 3:**
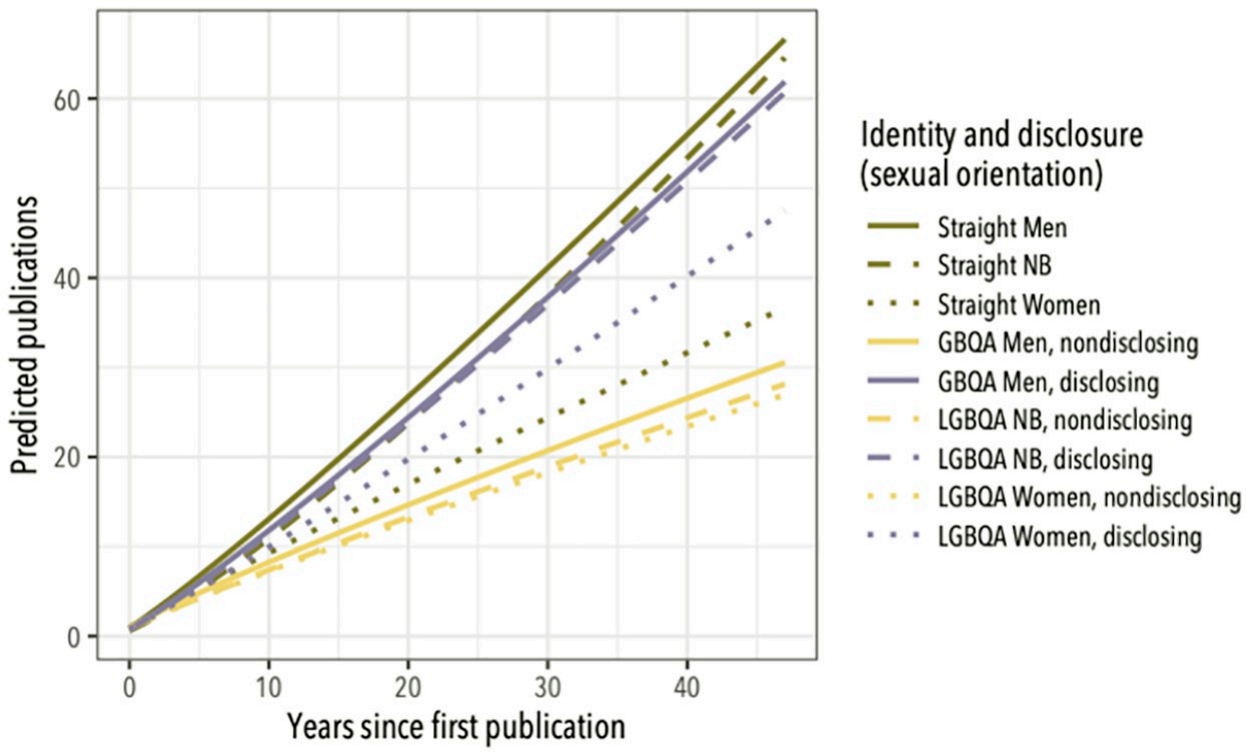
Predicted relationship between publication count and time since first publication for different LGBTQA identity/disclosure categories. From the best-fit model explaining publication count in the 2016 survey data (Tables 1 and 2). Color-coding of prediction lines follows Fig 1B.

The best-fit model predicts publication counts that increase with time since first publication, with the greatest rate of publication accumulation predicted for straight men, followed by straight nonbinary individuals, GBQA men who disclosed their sexual orientations, LGBQA women who disclosed their sexual orientations, straight women; and then by LGBQA participants who did not disclose their sexual orientation (Fig 3; Table 2). The model predicts that, 20 years after first beginning to publish peer-reviewed papers, straight men will have authored an average of 27 papers, disclosing queer men will have authored an average of 24 papers, and nondisclosing queer men will have authored 15. Among nonbinary scientists, straight individuals and disclosing LGBQA individuals are predicted to have authored about 24 papers in their first 20 years of publishing, while nondisclosing LGBQA individuals are predicted to have authored 13. Straight women are predicted to have authored about 17 papers in their first 20 years of publishing, while LGBQA disclosing women are predicted to have authored about 20 papers, and LGBQA nondisclosing women are predicted to have authored about 13.

**Table 2:** Parameter estimates for the best-fit model (Table 1).

Coefficient for	Estimate	Std. error	t-value	P (|T| > |t|)
(intercept)	-0.033	0.04972	-0.664	0.50699
Straight nonbinary	-0.18836	0.15437	-1.22	0.22255
Straight women	0.02958	0.0631	0.469	0.63923
Queer men, nondisclosing	0.026	0.07075	0.367	0.71332
Queer men, disclosing	-0.06596	0.07077	-0.932	0.35147
Queer nonbinary, nondisclosing	-0.04296	0.07604	-0.565	0.57217
Queer nonbinary, disclosing	-0.0846	0.1	-0.846	0.3977
Queer women, nondisclosing	-0.03311	0.06322	-0.524	0.60051
Queer women, disclosing	-0.07352	0.08025	-0.916	0.35974
Time since first publication	1.10436	0.05301	20.832	< 10^−6^
Straight nonbinary × time	0.10394	0.17001	0.611	0.54104
Straight women × time	-0.172	0.069	-2.493	0.01276
Queer men, nondisclosing × time	-0.21694	0.0859	-2.526	0.01164
Queer men, disclosing × time	0.02016	0.07896	0.255	0.79855
Queer nonbinary, nondisclosing × time	-0.19714	0.09775	-2.017	0.04387
Queer nonbinary, disclosing × time	0.02615	0.1124	0.233	0.81609
Queer women, nondisclosing × time	-0.21438	0.07564	-2.834	0.00464
Queer women, disclosing × time	-0.04379	0.0907	-0.483	0.62927

## Discussion

Longitudinal, observational, and survey-based studies have begun to delineate a pattern of systematic disparities in STEM careers between minoritized sexual and gender identities and straight, binary, cisgender identities [37, 49], similar to disparities seen between women and men, or between people of color and white individuals [34, 36, 50–52]. LGB-identified individuals are less likely than their straight peers to persist in STEM undergraduate majors [49, 53], and they are significantly underrepresented in STEM careers [37]; moreover, LGBT individuals working in STEM are more likely to report negative workplace experiences than non-LGBT colleagues [52]. Open expression or nondisclosure of LGBTQA identities in professional contexts provides a window into the underlying causes of these disparities since, on the one hand, nondisclosure is generally recognized as a source of stress and job dissatisfaction [20, 21], and on the other hand, LGBTQA individuals who describe their workplaces as having welcoming climates are more likely to disclose their identities at work [1, 47]. The data we present here identify a concrete productivity effect associated with disclosure or nondisclosure, and suggest how gender identity and expression may intersect with this effect.

In examining responses to the 2013 and 2016 Queer in STEM surveys, we find support for our hypotheses that (1) nondisclosure of queer identity is associated with reduced productivity in LGBTQA-identified scientists, that (2) productivity differences are better explained by nondisclosure reducing productivity than by productivity facilitating disclosure, and that (3) disclosure of gender identity and transgender status are not associated with productivity differences in the same way as disclosure of sexual orientation. Overall, these results are consistent with the belief that open expression of queer identities in the workplace promotes greater productivity [8, 26, 27, 29], and align with prior work showing the stressful impacts of nondisclosure [20, 23, 25, 26] and its effects on feelings about colleagues and employers [8, 27]. Our 2016 survey also finds lower publication rates for cisgender straight women, consistent with prior work documenting a productivity cost attributable to the systematic disadvantages women face in academic and scientific careers [31, 34, 36, 50]—and the productivity costs we find for nondisclosing LGBQA survey participants are comparable to the reductions found for cisgender straight women (Fig 3).

Our 2016 survey finds reductions in productivity reported by participants whose self-described gender expression is feminine or nonconforming to the gender binary (Fig 2D), which expands on our understanding of previously documented gender bias in STEM careers [34, 36, 50]. It also aligns with a possibility suggested by longitudinal and observational evidence that gay and bisexual men are less likely to persist in STEM undergraduate majors and less likely to be employed in STEM fields than straight men, while lesbian and bisexual women do not show reduced persistence or STEM employment rates relative to straight women [37, 49]. Although superficially suggesting contradictory outcomes for men and women who are sexual minorities, both of these observations are consistent with a broader pattern of privileging masculinity in STEM [47]. Our findings of reduced publication rates for women with masculine gender expression would also align with this pattern (Fig 2E), though this effect is not significant after correction for multiple testing.

Our data enriches understandings of gender-normative “heteroprofessionalism”, and its impact on workplace productivity and career advancement [29, 31, 47]. Because our 2016 survey treats openness about gender identity and cisgender or transgender status separately from openness about sexual orientation, we can see that the effect of disclosing a minority gender identity or transgender status may not neatly parallel the effect of disclosing a minority sexual orientation—though our survey design prevents us from clearly differentiating effects of gender modality and gender identity. This finding reflects the complexity of intersections among gender identity, gender modality, gender expression, and sexual orientation. One key way in which transgender identity can differ from minority sexual orientations is that transgender individuals who present as their true gender without disclosing transgender modality (“passing” or “going stealth”, in the terminology used by some members of the community) would not be expected to experience the dissonance or stress associated with nondisclosure of sexual orientation for cisgender gay men or cisgender lesbians—rather, this form of nondisclosure can represent social acceptance of a trans individual’s true gender identity [54]. In contrast, disclosing transgender identity directly confronts cisgender-heterosexist norms, and may create greater stress or tension with colleagues and coworkers. We posit that this may explain why disclosure of gender identity or transgender status is not associated with a significant increase in publication count for nonbinary or transgender participants (Fig 2C), even as feminine or nonconforming gender expression was associated with significant differences in publication counts for cisgender participants (Fig 2D and 2E). This highlights the importance of considering gender expression, gender identity, and gender modality relative to cultural expectations in a particular context, especially for interrogating the experiences of transgender, nonbinary, and gender nonconforming individuals.

While a positive relationship between productivity and disclosure of queer identity is consistent with the longstanding hypothesis that nondisclosure reduces productivity, in principle it could also be consistent with the alternative hypothesis that more accomplished individuals are more likely to feel secure enough to disclose minority identity and accept the attendant risks. However, the patterns in responses to our 2016 survey are, on balance, more consistent with the former hypothesis than the latter. First, if productivity enabled disclosure, we would expect that LGBQA participants who disclose their sexual orientation would have higher publication rates than straight participants as well as exceeding the publication rate of nondisclosing LGBQA participants; instead, we see that disclosing LGBQA participants have publication rates similar to straight participants (Fig 2A and 2B), suggesting that disclosing LGBQA participants are not unusually productive in comparison to their broader peer group. Second, if productivity enabled disclosure, we would expect to see that disclosing LGBQA participants achieve particular academic career stages more rapidly than their peers; but there is no difference in the time since first publication among disclosing LGBQA participants, nondisclosing LGBTA participants, and straight participants of the same career rank (S6 Fig). Taken together, these patterns are consistent with nondisclosure decreasing publication productivity, rather than LGBQA individuals waiting to disclose until they have achieved exceptional levels of productivity or job security relative to their peers. Methods such as cohort studies that track the productivity of LGBTQA-identified individuals and compare it to their choices of disclosure or nondisclosure over time would more directly address the causal relationship be, though recruiting long-term study participants who do not disclose queer identity in the workplace would likely be a substantial challenge.

We note that differences in publication rates seen in our survey results may not be exclusively attributable to disadvantages imposed by biases against queer identities or women in STEM, but may also reflect differing commitments to forms of scholarship beyond publication of peer-reviewed research articles. Members of minoritized groups in faculty positions may be called upon for more service and mentoring work as a result of their minoritized identities, but they may also put greater priority on that work as a result of their own experiences [35, 36, 55, 56]. Reducing disparities in retention and representation of LGBTQA-identified people in STEM careers may therefore require a broader understanding of how scholarly contributions and impact are evaluated [34].

We also note that our survey data capture conditions at two relatively narrow points in time (mid-2013, the second half of 2016) in a decade when the broader legal and social environment for LGBTQA-identifying individuals shifted dramatically in the United States, the region in which the overwhelming majority of survey participants lived—and that changes to these conditions are ongoing [10–12]. The publication records reported by survey participants, however, represent longer individual histories of experience, shaped by conditions pre-dating major legal victories for employment nondiscrimination protections, and we believe there is value in documenting those experiences. Moreover, formal legal protections are far from synonymous with acceptance and security in the interpersonal interactions of an individual’s workplace experience. A recognized legal right to sue for wrongful termination does not necessarily reduce tension with coworkers, or other subtle forms of discrimination [6], and, as has been well documented in the cases of other legally protected minoritized identities, it does not eliminate disparities in career outcomes [36, 50, 57]. These considerations mean that we have every reason to think the phenomena we see in surveys taken in 2013 and 2016 continue to operate in 2022; and indeed studies conducted more since the first publication of results from our 2013 survey [1] have generally replicated or elaborated on evidence of disadvantages for LGBTQA-identified individuals in STEM career paths [49, 51, 58].

Future research on queer identities in STEM and other workplaces would benefit from designs such as cohort studies and qualitative approaches that can directly address the diversity of personal experiences and reasons for disclosure or nondisclosure at the individual level. Our anonymous survey data necessarily provide only a view of broad patterns resulting from data collection and aggregation into simplified categories of identity and experience. Nevertheless, the patterns observable with this data align in striking ways with personal experiences recounted in smaller settings, and with predictions arising from psychological studies of nondisclosure. The observation that open expression of queer identity in the workplace is associated with accomplishment has implications for the quality and value of scientific careers beyond the experiences of sexual and gender-identity minorities—the same pattern may extend to other “invisible” minoritized or stigmatized statuses and identities, such as chronic disease, disabilities, or mental health conditions. We further predict that the effects of sexual and gender minority identities, and their disclosure, will interact with other minoritized identities such as race, ethnicity, and disability status, and we would suggest that focused examination of these interactions is an important direction for future work. We argue that the relationship that we find between productivity and expression of LGBTQA identities reinforces the need for greater support, at all levels of training and advancement, to make STEM careers accessible and sustainable for participants across the full range of human diversity.

## Materials and methods

### Data collection

#### 2013 survey

The 2013 Queer in STEM survey is described in detail in ref [1]. In brief, from 7 May to 31 July 2013, we asked LGBTQA-identified professionals in STEM fields to answer a 58-item online survey. The University of Minnesota Institutional Review Board approved the study in February 2013, and approved a change in protocol to allow for a larger-than-expected number of participants in June 2013 (project ID 1302E28561). We recruited participants via online social networks, e-mail listservs, and online forums for relevant STEM and LGBTQA organizations. Because of the potentially sensitive nature of the survey’s topics—particularly asking about identities that individuals may not disclose in professional settings—the survey was conducted anonymously. Informed consent was ensured with a disclosure statement presented to participants prior to beginning the survey, which clearly stated that completing the survey in full would be understood to mean participants consented to the use of their response in our analysis, and that participants could exit the survey at any time prior to final submission to have their responses up to that point excluded from analysis. As our focal population was individuals working in STEM careers, we did not recruit minor participants, and deleted survey responses from any participant indicating they were younger than 18 years old.

We asked participants to provide the number of peer-reviewed papers they had published; in a handful of cases in which a participant gave a range (e.g., “more than 20”) we used the lowest value in that range. We asked participants to rate their disclosure of LGBTQ identity to colleagues in the same department or division of their institution, and to colleagues in other departments or divisions, on a numeric scale from 0 (“I am not out to anyone in this group”) to 5 (“As far as I’m aware, everyone in this group could know”). These ratings were strongly and positively correlated (correlation = 0.88, P < 0.001), with participants most likely to describe themselves as either entirely out to colleagues (averaged openness ratings of 5) or not out at all (averaged ratings of 0; ref [1]). For the present analysis, we classified participants as “nondisclosing” if their averaged rating was 2.5 or lower, and “disclosing” otherwise.

We asked participants to describe their gender identity and cisgender or transgender status, and classified the gender identity of cisgender and transgender participants as “men” or “women” if they used only these terms to describe themselves, or “nonbinary” if they used terms indicating identities beyond the man/woman binary, including “non-binary”, “gender-queer”, “gender fluid”, “gender non-conforming”, or “agender”. Finally, we asked participants to describe their current job position or career stage. A total of 633 participants from the 2013 survey who indicated current employment in academic STEM fields and who reported having published at least one peer-reviewed paper are included in the present analysis (S1 Table).

#### 2016 survey

The 2016 Queer in STEM survey mirrored that of the 2013 Queer in STEM survey was developed as a follow-up to the 2013 study, and its planning and execution followed that prior work in many respects. Notable changes included separate treatment of participants’ disclosure of their sexual orientation and their gender identities or modalities, and the addition of responses from non-LGBTQA participants. The California State University Los Angeles Institutional Review Board approved the proposed survey items and study design in 2016, (project ID 844053–1). We recruited participants to take the new online survey via online social networks (Twitter and Facebook), e-mail listservs for STEM professional organizations, and online forums for relevant STEM and LGBTQA organizations. Following a “snowball sampling” approach, we also asked participants in the survey to pass the survey link along to colleagues or other acquaintances who could also participate. The online survey was open from 20 June to 25 December 2016. As in the 2013 survey, participation in the 2016 survey was anonymous. Informed consent was ensured with a disclosure statement presented to participants prior to beginning the survey, which clearly stated that completing the survey in full would be understood to mean participants consented to the use of their response in our analysis, and that participants could exit the survey at any time prior to final submission to have their responses up to that point excluded from analysis. As our focal population was individuals working in STEM careers, we did not recruit minor participants, and deleted survey responses from any participant indicating they were younger than 18 years old.

To address productivity, we included an item asking for the time elapsed since participants’ first publication as a control for seniority (“How many years have passed since publication of your first peer-reviewed publication?”; full item text is provided in the S1 File) in addition to an item asking for their publication count (“To date, how many manuscripts have you published in peer-reviewed journals?”).

We asked participants to describe their sexual orientation, their gender identity and cisgender or transgender status, and their gender expression, providing common terms as well as an open-ended text entry for each (see S1 File). For analysis, we classified participants as “LGBQA” (for women or nonbinary participants) or “GBQA” (for men) if they described their sexual orientation using any terms besides “straight”. We classified cisgender and transgender participants’ gender identities as “men” or “women” if they used those terms to describe themselves, and as “nonbinary” if they selected or wrote in identities beyond the man/woman binary, including “non-binary”, “gender-queer”, “gender fluid”, “gender non-conforming”, or “agender”. Finally, we classified cisgender and transgender participants’ gender expressions as “masculine” or “feminine” if they used only those terms to describe their gender expression, and as “nonconforming” if they selected “androgynous” or wrote in other terms to describe their gender expression.

We then asked participants to rate their disclosure of their sexual orientation and their gender identity and cisgender or transgender status to different groups in professional academic contexts: “Professors at your institution”, “Lab assistants and institutional staff”, “Advisees and/or graduate students”, and “Undergraduate students” on a six-point scale from “As far as I am aware, no one in this group knows” to “As far as I am aware, everyone in this group knows”, with an option to select “N/A” if participants did not interact with any particular group in a professional context. Following the approach used in the 2013 study [1] we converted disclosure scores for sexual orientation and for gender identity and cisgender or transgender status to numeric values from 1 to 6, and averaged them across groups, then classified queer participants as “nondisclosing” if their averaged rating was at or below the midpoint value of 3, and “disclosing” otherwise.

We filtered 3,884 full survey responses to remove 201 participants from the dataset who were undergraduate students (97 respondents), non-STEM workers (85 respondents), or under 20 years of age (19 respondents). Of the remaining 3,683 participants, 2,455 (67%) indicated a current position within academia and 1,228 (33%) within industry. Of the same 3,683 total participants, 2,465 (67%) identified in some way under the LGBTQA umbrella and 1,218 respondents (33%) identified as cisgender straight (i.e. non-LGBTQA). We also removed from analysis four participants in the 2016 survey reporting exceptionally high publication counts (> 300 papers).

We further filtered to include only participants who described themselves as working in an academic setting and having published at least one peer-reviewed paper, resulting in a total of 1,745 participant responses in the present analysis (S1 Table); of these, 1,116 identified as LGBTQA and 629 as straight and cisgender (S2 Table). LGBTQA participants did not significantly differ from straight cisgender participants in their distribution among major STEM fields (S2 Table; Χ^2^_df=8_ test, p = 0.17). However, the two groups did differ significantly in their distribution among academic position types (S2 Table; Χ^2^_df=9_ test, p < 10^−6^), and in their time since first publication, with LGBTQA participants having spent less time publishing (S2 Table; mean ± SE = 6.2 ± 0.2 years since first publication for LGBTQA participants, 8.6 ± 0.4 years for straight cisgender participants; Wilcoxon sign-rank test p < 10^−6^).

### Statistical analyses

We conducted all analysis in R, version 4.0 [59]. We restricted analysis to survey participants who identified themselves as currently working in academic settings and who reported at least one peer-reviewed publication. We tested for differences in log-transformed publication count among groupings based on queer identities, disclosure statuses, gender expression, STEM fields, and academic positions using two-sided t-tests (via the t.test() function) when testing for differences between two groups, or one- or two-way ANOVA (the aov() function) when testing among more than two groups. When ANOVA testing found significant among-grouping differences in publication count, we identified significant differences in pairwise comparisons among groupings using Tukey honest significant difference testing for nonzero between-group differences, using the TukeyHSD() function. In the 2016 data, we tested for a relationship between publication count and time since first publication using the cor.test() function to estimate the product-moment correlation between the base-10 logarithm of publication count and the base-10 logarithm of years since first publication plus 1. We compared explanatory power for all variables showing significant associations to publication count by fitting linear models to log-transformed publication counts with the lm() function, then comparing the fit of alternative models in terms of corrected Akaike information criterion (AICc) scores [48], calculated using the AICc() function provided in the MuMIn package [60].

## Supporting information

S1 Fig Publication counts and workplace climate in the 2013 survey. Publication counts stratified by participants’ ratings of their workplace’s climate for LGBTQA individuals, in the 2013 survey. Differences among workplace ratings are nonsignificant (one-way ANOVA on log-transformed data, p = 0.11).
(PDF)

S2 Fig Publication counts and STEM fields in the 2013 survey. Publication counts stratified by participants’ STEM fields, in the 2013 survey. Differences among fields are nonsignificant (one-way ANOVA on log-transformed data, p = 0.13).
(PDF)

S3 Fig Job position and disclosure of queer identity in the 2013 survey. Participants in the 2013 survey, binned by academic position description (in rough order of seniority, bottom to top) and whether they disclosed LGBTQA identity in professional settings (purple) or did not disclose queer identity (yellow). Disclosure is unevenly distributed among position types (chi-squared test, p < 10^−5^), with larger proportions of participants who did not disclose queer identities in less-senior positions.
(PDF)

S4 Fig Publication counts and workplace climate in the 2016 survey. Differences among workplace ratings are nonsignificant (one-way ANOVA on log-transformed data, p = 0.21).
(PDF)

S5 Fig Publication counts and STEM fields in the 2016 survey. Differences among workplace ratings are nonsignificant (one-way ANOVA on log-transformed data, p = 0.80).
(PDF)

S6 Fig Time since first publication by academic career stage in the 2016 survey. Time since first publication stratified by identity, disclosure status, and academic career stage, for 1,424 participants at these career stages in the 2016 survey. Differences among identity and disclosure groupings within each career stage are nonsignificant (Tukey HSD, p > 0.05 in all cases).
(PDF)

S1 Table Summary of the 2013 and 2016 survey data. (DOCX)

S2 Table Comparison of LGBTQA and cisgender straight participants in the 2016 survey. (DOCX)

S1 File Supplementary methods. (DOCX)
