# Supplementary figures and images for: Nondisclosure of queer identities is associated with reduced scholarly publication rates
Source: PLoS One. 2022 Mar 2;17(3):e0263728. doi: 10.1371/journal.pone.0263728 (PMC8890643; doi:10.1371/journal.pone.0263728)

Workplace climate rating

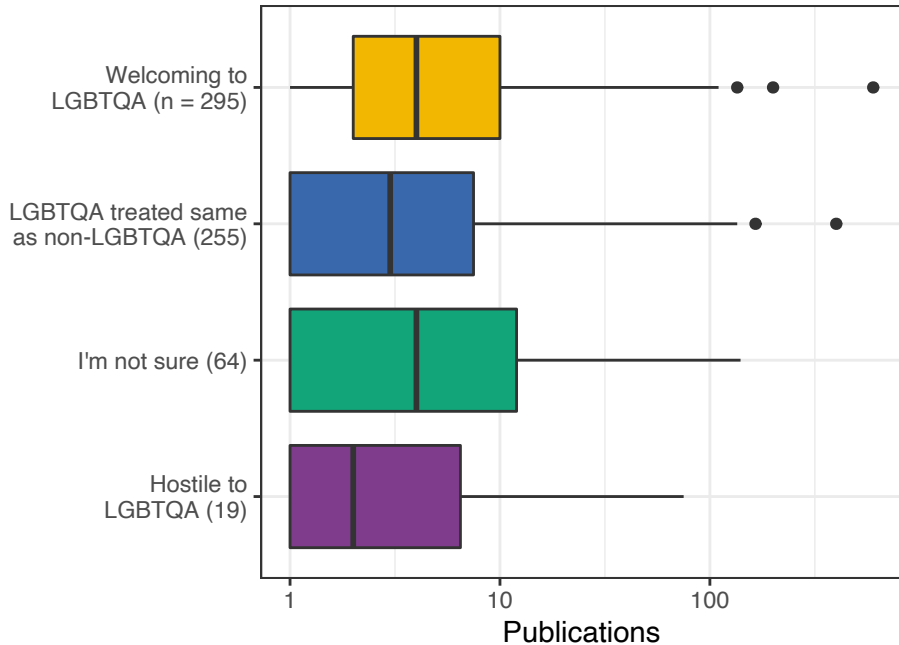

Supplement: S1 Fig — Publication counts stratified by participants’ ratings of their workplace’s climate for LGBTQA individuals, in the 2013 survey. Differences among workplace ratings are nonsignificant (one-way ANOVA on log-transformed data, p = 0.11). (PDF) [file pone.0263728.s001.pdf]

STEM field

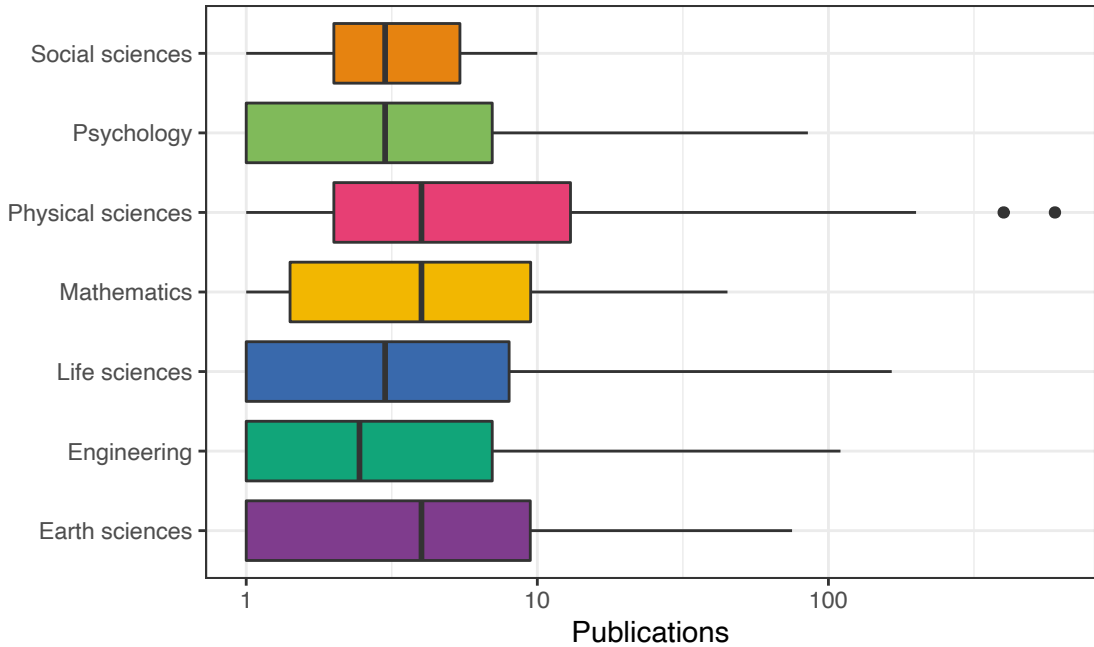

Supplement: S2 Fig — Publication counts stratified by participants’ STEM fields, in the 2013 survey. Differences among fields are nonsignificant (one-way ANOVA on log-transformed data, p = 0.13). (PDF) [file pone.0263728.s002.pdf]

Position

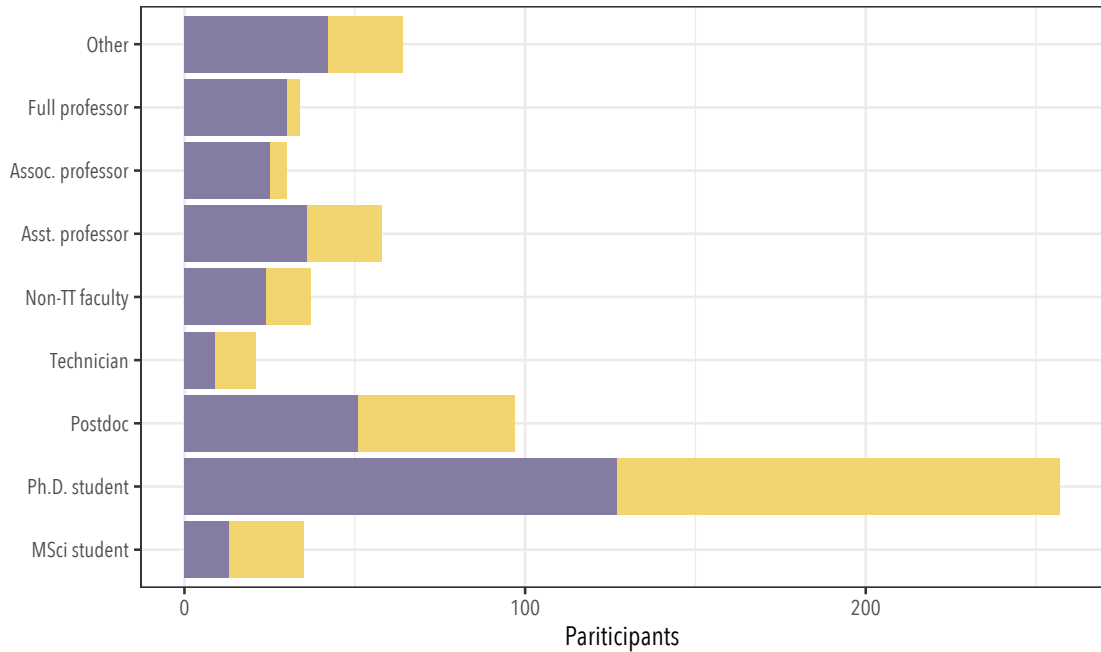

Supplement: S3 Fig — Participants in the 2013 survey, binned by academic position description (in rough order of seniority, bottom to top) and whether they disclosed LGBTQA identity in professional settings (purple) or did not disclose queer identity (yellow). Disclosure is unevenly distributed among position types (chi-squared test, p < 10−5), with larger proportions of participants who did not disclose queer identities in less-senior positions. (PDF) [file pone.0263728.s003.pdf]

# Workplace climate rating

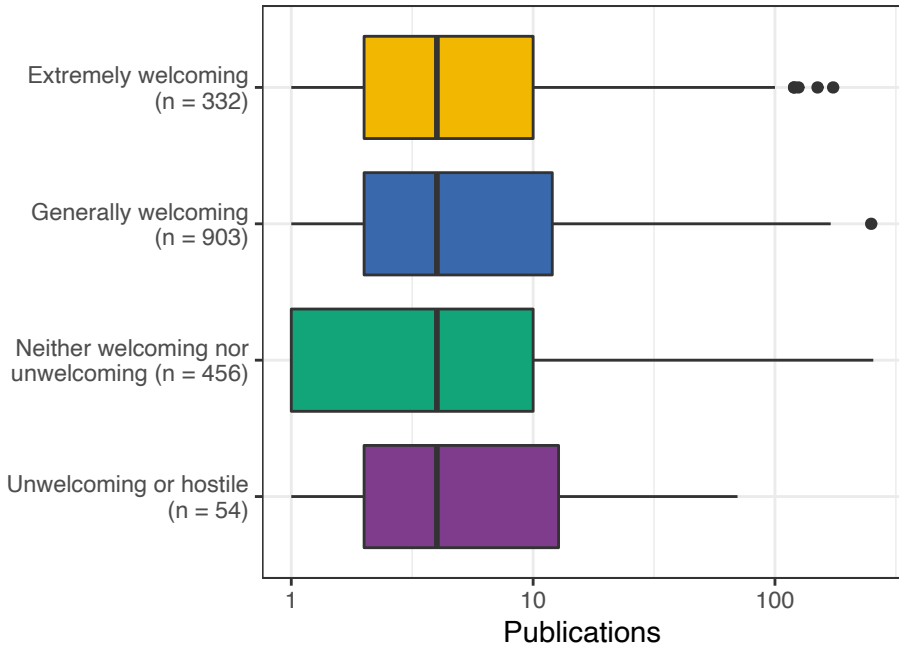

Supplement: S4 Fig — Differences among workplace ratings are nonsignificant (one-way ANOVA on log-transformed data, p = 0.21). (PDF) [file pone.0263728.s004.pdf]

STEM field

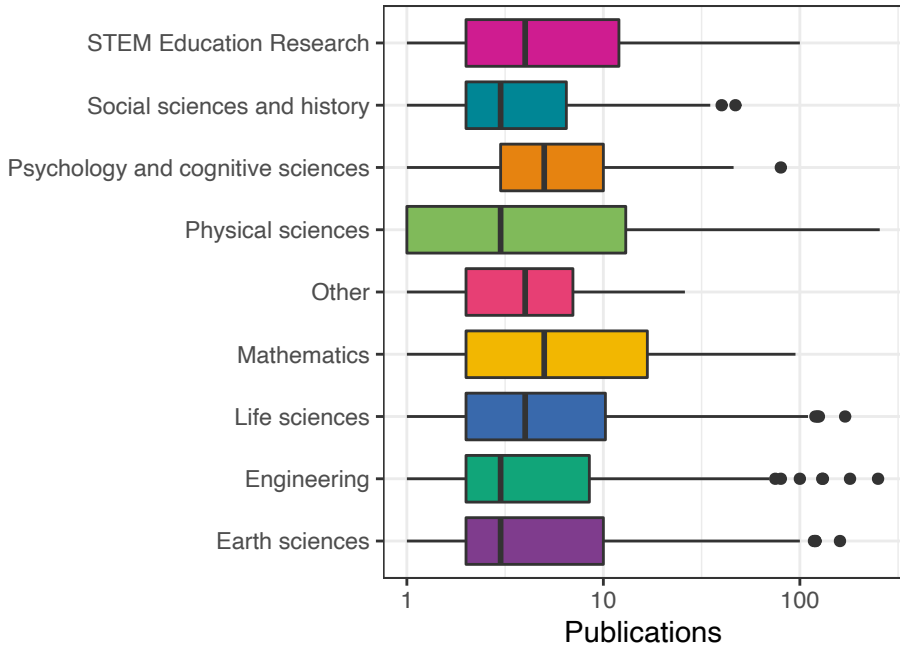

Supplement: S5 Fig — Differences among workplace ratings are nonsignificant (one-way ANOVA on log-transformed data, p = 0.80). (PDF) [file pone.0263728.s005.pdf]

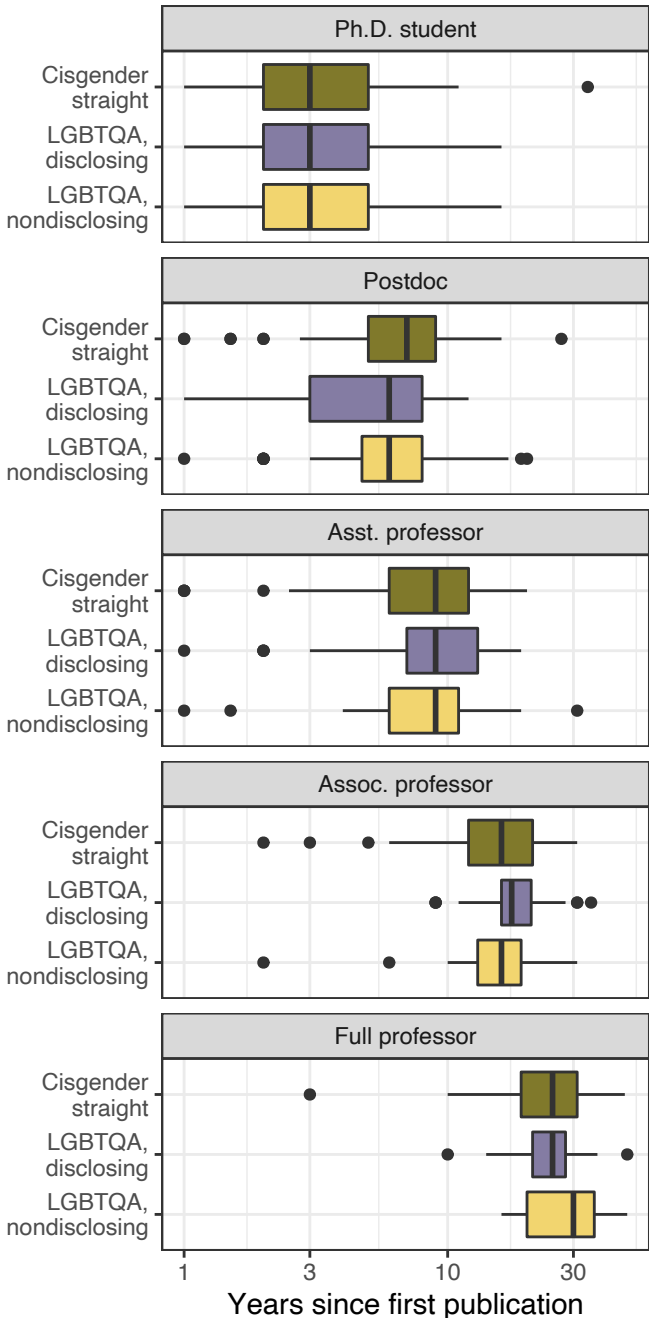

Supplement: S6 Fig — Time since first publication stratified by identity, disclosure status, and academic career stage, for 1,424 participants at these career stages in the 2016 survey. Differences among identity and disclosure groupings within each career stage are nonsignificant (Tukey HSD, p > 0.05 in all cases). (PDF) [file pone.0263728.s006.pdf]
