# Supplementary material for: Nondisclosure of queer identities is associated with reduced scholarly publication rates
Source: PLoS One. 2022 Mar 2;17(3):e0263728. doi: 10.1371/journal.pone.0263728 (PMC8890643; doi:10.1371/journal.pone.0263728)
Supplement: S1 Table — (DOCX) [file pone.0263728.s007.docx]

S1 Table. Summary of the 2013 and 2016 survey data.

| **Grouping** | **2013 survey** | | **2016 survey** | |
| --- | --- | --- | --- | --- |
| Total participants | 633 | (100%) | 1,745 | (100%) |
| *Sexual orientation and gender identity* |  |  |  |  |
| Queer women | 287 | (45%) | 443 | (25%) |
| Queer nonbinary | 62 | (10%) | 216 | (12%) |
| Queer men | 284 | (45%) | 438 | (25%) |
| Straight women | - |  | 378 | (22%) |
| Straight nonbinary | - |  | 23 | (1%) |
| Straight men | - |  | 247 | (14%) |
| *Gender identity and gender modality* |  |  |  |  |
| Transgender women | 15 | (2%) | 19 | (1%) |
| Transgender nonbinary | 14 | (2%) | 176 | (10%) |
| Transgender men | 7 | (1%) | 17 | (1%) |
| Cisgender women | 272 | (43%) | 802 | (46%) |
| Cisgender nonbinary | 48 | (8%) | 63 | (4%) |
| Cisgender men | 277 | (44%) | 668 | (38%) |
| *Gender modality and sexual orientation* |  |  |  |  |
| Transgender, queer | 36 | (5%) | 193 | (11%) |
| Transgender, straight | - |  | 19 | (1%) |
| Cisgender, queer | 597 | (94%) | 904 | (52%) |
| Cisgender, straight | - |  | 629 | (36%) |
| *STEM field* |  |  |  |  |
| Earth sciences | 42 | (7%) | 174 | (10%) |
| Engineering | 68 | (11%) | 199 | (11%) |
| Life sciences | 322 | (51%) | 720 | (41%) |
| Mathematics | 43 | (7%) | 66 | (4%) |
| Physical sciences | 121 | (19%) | 393 | (23%) |
| Psychology | 23 | (4%) | 74 | (4%) |
| Social sciences | 14 | (2%) | 39 | (2%) |
| Others | - |  | 80 | (5%) |
| *Current position* |  |  |  |  |
| Master’s student | 35 | (6%) | 72 | (4%) |
| PhD student | 257 | (41%) | 715 | (41%) |
| Postdoctoral researcher | 97 | (15%) | 263 | (15%) |
| Technician | 21 | (3%) | 102 | (6%) |
| Non-tenure-track or adjunct faculty | 37 | (6%) | 110 | (6%) |
| Assistant professor | 58 | (9%) | 196 | (11%) |
| Associate professor | 30 | (5%) | 135 | (8%) |
| Full professor | 34 | (5%) | 115 | (7%) |
| Others | 64 | (10%) | 37 | (2%) |
| *Disclosure status* |  |  |  |  |
| LGBTQA identity disclosed | 357 | (56%) | - |  |
| not disclosed | 276 | (44%) | - |  |
| Sexual orientation disclosed | - |  | 457 | (26%) |
| not disclosed | - |  | 659 | (38%) |
| disclosure N/A | - |  | 629 | (36%) |
| Gender ID or trans status disclosed | - |  | 70 | (4%) |
| not disclosed | - |  | 142 | (8%) |
| disclosure N/A | - |  | 1,533 | (88%) |
