# Supplementary material for: Nondisclosure of queer identities is associated with reduced scholarly publication rates
Source: PLoS One. 2022 Mar 2;17(3):e0263728. doi: 10.1371/journal.pone.0263728 (PMC8890643; doi:10.1371/journal.pone.0263728)
Supplement: S2 Table — (DOCX) [file pone.0263728.s008.docx]

S2 Table. Comparison of LGBTQA and cisgender straight participants in the 2016 survey

| **Grouping** | **LGBTQA** | | **Cisgender straight** | |
| --- | --- | --- | --- | --- |
| Total | 1,116 | (100%) | 629 | (100%) |
| *STEM field* |  |  |  |  |
| Earth sciences | 106 | (9%) | 68 | (11%) |
| Engineering | 125 | (11%) | 74 | (12%) |
| Life sciences | 482 | (43%) | 238 | (38%) |
| Mathematics | 44 | (4%) | 22 | (3%) |
| Physical sciences | 240 | (22%) | 153 | (24%) |
| Psychology | 50 | (4%) | 24 | (4%) |
| Social sciences | 23 | (2%) | 16 | (3%) |
| Others | 14 | (1%) | 4 | (>1%) |
| *Current position* |  |  |  |  |
| Master’s student | 58 | (5%) | 14 | (2%) |
| PhD student | 502 | (45%) | 213 | (34%) |
| Postdoctoral researcher | 161 | (10%) | 102 | (16%) |
| Technician | 77 | (7%) | 25 | (4%) |
| Non-tenure-track or adjunct faculty | 39 | (3%) | 35 | (6%) |
| Assistant professor | 104 | (9%) | 92 | (15%) |
| Associate professor | 71 | (6%) | 64 | (10%) |
| Full professor | 54 | (5%) | 61 | (10%) |
| Others | 27 | (2%) | 10 | (2%) |
| *Time since first publication (years)* |  |  |  |  |
| Mean ± standard error | 6.2 ± | 0.2 | 8.6 ± | 0.4 |
| Median (95% density interval) | 4 | (0, 27) | 6 | (0, 33) |
