# Supplementary material for: Nondisclosure of queer identities is associated with reduced scholarly publication rates
Source: PLoS One. 2022 Mar 2;17(3):e0263728. doi: 10.1371/journal.pone.0263728 (PMC8890643; doi:10.1371/journal.pone.0263728)
Supplement: S1 File — (DOCX) [file pone.0263728.s009.docx]

Supporting Methods for

**Nondisclosure of queer identities is associated with reduced scholarly publication rates**

Joey Nelson, Allison Mattheis, and Jeremy B. Yoder^*^

*Corresponding author. Email: jeremy.yoder@csun.edu

**2013 survey**

The present analysis examines responses from 633 participants in the 2013 Queer in STEM survey [1] who self-identified as working in academia at any level from graduate school to retirement, and who reported having authored at least one peer-reviewed paper.

The 2013 Queer in STEM survey is described in detail in ref [1]. In brief, from 7 May to 31 July 2013, we asked LGBTQA-identified professionals in STEM fields to answer a 58-item online survey. The University of Minnesota Institutional Review Board approved the study in February 2013, and approved a change in protocol to allow for a larger-than-expected number of participants in June 2013 (project ID 1302E28561). We recruited participants via online social networks (Twitter and Facebook), e-mail listservs, and online forums for relevant STEM and LGBTQA organizations.

Here, we analyze responses from survey items addressing (1) the number of peer-reviewed papers participants had authored, (2) their self-evaluation of disclosure of queer identity in workplace contexts, (3) their gender identity and cisgender or transgender status, (4) their rating of their workplace climate for queer individuals, and (5) their job position or career stage. For each of these items, we give the text of the item as presented in the original online survey, the multiple-choice options offered for responses.

**Publication counts.**

*Item text*: To date, how many manuscripts have you had accepted for peer­reviewed publication, as a coauthor at any rank? Please enter a number in the space below.

*Responses offered:*

- [Text entry]

**Queer identity disclosure in workplace contexts.**

*Item text:* Use the following scale to describe how “out” and open you are to various groups of people with whom you interact.

*Ratings requested for:*

- Coworkers/colleagues in your department or division [0,1,2,3,4,5]
- Coworkers/colleagues in departments or divisions beyond your own [0,1,2,3,4,5]

*Responses offered:*

0: I am not out to anyone in this group.

1: A few people in this group know my orientation or identity.

2: Fewer than half the people in this group know.

3: Most people in this group know.

4: Almost everyone in this group knows.

5: As far as I'm aware, everyone in this group could know.

**Gender identity and cisgender or transgender status.**

*Item text:* How do you describe your gender identity? Please choose all that apply, or provide an alternate term in the space provided.

*Responses offered*:

- Female
- Male
- Transgender
- Androgynous
- Genderqueer
- Other: [text entry]

**Workplace climate**

*Item text:* Based on your experiences, would you characterize the social climate of the institution at which you study or work as generally welcoming or generally hostile to LGBTQ people?

*Responses offered*:

- The climate is welcoming
- LGBTQ people are treated the same as non-LGBTQ people
- The climate is hostile
- I’m not sure

**Job position or career stage.**

*Item text:* What is the best description of your current role or job position? Please select ONE:

*Responses offered:*

- MSci student
- Ph.D. student
- Laboratory/research technician
- Laboratory manager
- Research associate/research scientist
- Postdoctoral researcher
- Adjunct faculty/lecturer
- Assistant professor
- Associate professor
- Full professor
- Other academic or research
- Other [text entry]

**2016 survey**

The present analysis examines responses from 1745 participants in the 2016 Queer in STEM survey who self-identified as working in academia at any level from graduate school to retirement, and who reported having authored at least one peer-reviewed paper. We analyze responses from survey items addressing (1) the number of peer-reviewed papers participants had authored, (2) the years elapsed since publication of their first paper, (3) their self-description of sexual orientation, (4) their self-evaluation of disclosure of sexual orientation in workplace contexts, (5) their gender identity and cisgender or transgender status, and (6) their self-evaluation of disclosure of gender identity and cisgender or transgender status in workplace contexts, (7) their gender, and (8) the climate of their workplace for LGBTQA identities. For each item, we give the text of the item and offered responses.

**Publication counts.**

*Item text:* To date, how many manuscripts have you published in peer‐reviewed journals?

Please enter a number in the space below.

*Responses offered:* [text entry]

**Years since first publication.**

*Item text:* How many years have passed since publication of your first peer‐reviewed publication? Please enter a number in the space provided, if applicable. If this is not part of your professional responsibilities or you have yet to publish, please leave blank.

*Responses offered:* [text entry]

**Sexual orientation.**

*Item text:* How do you describe your sexual orientation? Pick as many as desired from the following terms, or select “other.” If you prefer, you may provide an alternate term/description in the space provided. (Sexual Orientation: A person's sexual identity in relation to the gender identity/identities to which they are attracted.)

*Responses offered:*

- Asexual, Aromantic
- Asexual, Non-aromantic
- Bisexual
- Gay
- Lesbian
- Pansexual
- Queer
- Questioning
- Straight
- Other [text entry]

**Disclosure of sexual orientation.**

*Item text:* How aware of your current sexual orientation are the following groups of people? Please use the scale below to select the best answer or select N/A for “Not Applicable” if the group is not one with whom you interact.

*Ratings requested for:*

- Professors at your institution
- Lab assistants and institutional staff
- Advisees and/or graduate students
- Undergraduate students

*Responses offered*:

- As far as I am aware, no one in this group knows
- A few people in this group know
- Less than half of people in this group know
- Most people in this group know
- Almost everyone in this group knows
- As far as I am aware, everyone in this group knows
- N/A

**Gender identity and cisgender or transgender status.**

*Item text:* How do you describe your gender identity? Pick as many as desired from the following terms. If you prefer, you may provide an alternate term/description in the space provided. (Gender Identity: One's innermost concept of self as man, woman, a blend of both or neither – how individuals perceive themselves and what they call themselves. One's gender identity can be the same or different from their sex assigned at birth. Cisgender is a term applied to those whose gender identity matches the sex assigned to them at birth, whereas other terms below apply to those whose gender identity does not match the sex they were assigned at birth.)

*Responses offered:*

- Agender
- Cisgender Man
- Cisgender Woman
- Gender Fluid
- Gender Non-conforming
- Genderqueer
- Non-binary
- Trans
- Transgender Man (FTM)
- Transgender Woman (MTF)
- Other [text entry]

**Disclosure of gender identity and cisgender or transgender status.**

*Item text:* How aware of your current gender identity are the following groups of people?

Please use the scale below to select the best answer or select N/A for “Not Applicable” if the group is not one with whom you interact.

*Ratings requested for:*

- Professors at your institution
- Lab assistants and institutional staff
- Advisees and/or graduate students
- Undergraduate students

*Responses offered*:

- As far as I am aware, no one in this group knows
- A few people in this group know
- Less than half of people in this group know
- Most people in this group know
- Almost everyone in this group knows
- As far as I am aware, everyone in this group knows
- N/A

**Gender expression.**

*Item text:* How do you describe your gender expression? Pick as many as desired from the following terms. If you prefer, you may provide an alternate term/description in the space provided. (Gender Expression: External appearance of one's gender identity, usually expressed through behavior, clothing, haircut or voice, and which may or may not conform to socially defined behaviors and characteristics typically associated with being either masculine or feminine.)

*Responses offered:*

- Androgynous
- Feminine
- Masculine
- Other [text entry]

**Workplace climate**

*Item text:* Based on your experiences, how would you characterize the social climate for LGBTQ individuals where you work/study?

*Responses offered*:

- Extremely welcoming
- Generally welcoming
- Neither welcoming nor unwelcoming
- Unwelcoming or hostile
